# Unraveling the role of PIWI/piRNAs in stem cell therapy: Epigenetic mechanisms and therapeutic potentials

**DOI:** 10.1016/j.gendis.2025.101854

**Published:** 2025-09-18

**Authors:** Ying Yang, Xiangping Luo, Ermao Li, Zhengmao Li, Feiyan Zou, Jiayang Liao, Yizhi Wu, Bo Wei

**Affiliations:** a Institute of Translational Medicine, School of Basic Medicine, Department of Special Medicine, School of Public Health, Hengyang Medical College, University of South China, Heneyang Central Hospital, Hengyang, Hunan 42100l, China; b Department of Orthopaedics Trauma, The Second Affiliated Hospital of University of South China, Hengyang, Hunan 421001, China

**Keywords:** Epigenetic regulation, piRNA, PIWI proteins, PIWI/piRNA complexes, Stem cell therapy

## Abstract

The therapeutic potential of stem cell therapy critically depends on precise epigenetic regulation mediated by PIWI/piRNA complexes. This review synthesizes current literature to analyze the structure, interaction mechanisms, and epigenetic functions of PIWI proteins and their associated piRNAs (24–32 nt non-coding RNAs), with emphasis on stem cell therapy and disease modeling applications. PIWI/piRNA complexes critically regulate transcriptional control, chromatin remodeling, and stem cell plasticity. These complexes maintain stemness while promoting differentiation, serving as key biomarkers and therapeutic targets in cancers and skeletal disorders. However, translational challenges regarding specificity, delivery stability, and safety profiles persist. Advancing PIWI/piRNA-based therapeutics requires innovative technologies and expanded clinical validation to develop safe, targeted stem cell treatments for diverse pathologies.

## Introduction

Regenerative medicine is an interdisciplinary field that combines engineering and life sciences to develop technologies capable of restoring, maintaining, or enhancing living tissues and organs. Its fundamental goal is to create functional tissues that can replace missing organs or restore tissue functions that the organism cannot regenerate under physiological conditions.^1^ It relies on two main pillars: tissue engineering and stem cell therapy. Tissue engineering is an emerging research field involving novel strategies to enhance the repair capacity of damaged tissues,^2^ providing cells with a three-dimensional growth environment. Stem cells, found in both animal embryos and adult tissues, are a unique class of undifferentiated cells known for their abilities to self-renew, maintain an undifferentiated state, and exhibit proliferative capabilities. These cells are classified based on their origin, differentiation potential, and other distinctive properties.^3^ Stem cell therapy harnesses these cells and their derivatives to restore impaired functions and repair damaged tissues. Stem cell-based tissue engineering holds broad promise in neuronal repair, cardiac patches, skin regeneration, gene therapy, and cartilage tissue engineering.^4^ With advancements in medical science, the use of stem cells in treating a wide range of diseases has grown, highlighting the importance of fully understanding the unique characteristics and benefits of different stem cell types to achieve successful therapeutic outcomes.^5^

The combined application of stem cell therapy with exogenous gene expression is an emerging research approach, utilizing viral and non-viral vectors to deliver DNA/RNA genetic information into cells.^6^ To enhance therapeutic efficacy, it can also be integrated with tissue engineering approaches, including nanomaterials and 3D printing.^7^, ^8^, ^9^ RNA interference (RNAi) technology, a novel therapeutic invention and a molecular-level precision regulatory tool, enhances the accuracy and efficiency of stem cell therapy and tissue engineering. Small regulatory RNAs (sRNAs) primarily comprise three major categories: microRNAs (miRNAs), small interfering RNAs (siRNAs), and PIWI-interacting RNAs (piRNAs), which guide sequence-specific regulation of gene expression through RNAi. Furthermore, leveraging the specificity of sRNAs, RNAi can target any specific gene and reveal its roles in development, metabolism, aging, stress responses, and other critical processes.^10^^,^^11^ It is closely linked to biomaterials, stem cells, molecular imaging, gene therapy methods, and regenerative medicine.^1^^,^^4^^,^^12^

Epigenetics focuses on the study of gene expression changes that do not alter the DNA sequence but result from chemical modifications in DNA and associated proteins. These epigenetic mechanisms play a crucial role in regulating gene expression, cell differentiation, tissue development, and disease susceptibility,^13^ primarily through DNA methylation, histone modifications, chromatin remodeling, and the regulation of non-coding RNAs.^14^ In stem cells, differentiation is governed by selective gene expression driven by specific chromosomal and DNA modifications that are essential for regulating functions such as self-renewal and lineage differentiation. Epigenetic modifications are vital in maintaining pluripotency by repressing the expression of differentiation genes.^15^ Furthermore, histone modifications, which influence DNA transcription related to the cell cycle, can control stem cell aging, potentially leading to stem cell dysfunction and exhaustion. This understanding provides valuable therapeutic strategies for managing aging-related diseases.^16^

Initially believed to be restricted to germ cells, PIWI proteins and their cognate piRNAs have now been identified in somatic cells, where they play pivotal roles in stem cell therapy and epigenetic regulation. As members of the PAZ-PIWI domain (PPD) family within the Argonaute protein superfamily, PIWI proteins are indispensable for piRNA biogenesis and transposon silencing—critical processes for lineage development and cellular identity. The human Argonaute family comprises two subfamilies: Ago and PIWI. The PIWI subfamily includes four primary members: PIWIL1 (HIWI), PIWIL2 (HILI), PIWIL3 (HIWI3), and PIWIL4 (HIWI2), all sharing conserved structural and functional domains.^17^ piRNAs represent the largest class of non-coding RNAs, typically 24–32 nucleotides in length, and are characterized by a 5′ monophosphate uridine and a 2′-O-methylated 3′ terminus. They originate predominantly from genomic clusters rich in transposable elements (TEs)—mobile genetic elements capable of causing mutations through genomic repositioning.^18^ Unlike other small RNAs, piRNAs lack a defined secondary structure, are generated independently of Dicer from single-stranded piRNA cluster precursors, and undergo specific 3′ end 2′-O-methylation catalyzed by the Hen1 methyltransferase.^19^, ^20^, ^21^ These mature piRNAs specifically bind to PIWI proteins, forming functional complexes essential for diverse physiological functions.

The PIWI/piRNA complex not only serves as a key effector of RNAi but also achieves persistent reprogramming of stem cell genome stability maintenance, transposon silencing, and differentiation programs by inducing epigenetic modifications. This completes a functional loop: tissue engineering provides the physical scaffold, stem cells contribute the cellular source, and the piRNA/PIWI complex enables specific regulation through epigenetic mechanisms, collectively driving the cross-scale transformation of regenerative medicine from molecular intervention. Therefore, understanding the roles and mechanisms of PIWI/piRNAs is vital as they are implicated in disease development and provide novel targets and strategies for therapeutic interventions. A thorough comprehension of these functions and interactions is fundamental, offering essential theoretical support for future in-depth research and clinical trials.

## Biological and epigenetic mechanisms of PIWI/piRNAs

### The structure and function of PIWI proteins

**Figure 1:**
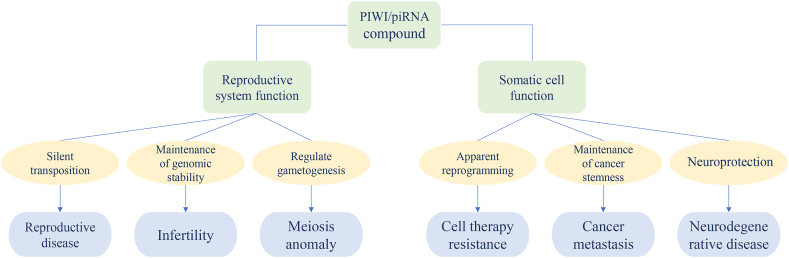
A roadmap for understanding Table 1.

PIWI proteins are structurally characterized by three key domains: a variable N-terminal region, central PAZ and MID domains, and a C-terminal PIWI domain.^22^ The PAZ domain specifically recognizes the 3′ ends of piRNA precursors, while the MID domain binds to their 5′ ends, collectively enabling precise piRNA interactions.^23^ The C-terminal PIWI domain exhibits RNase H-like nuclease activity essential for cleaving target RNAs guided by small non-coding RNAs (sncRNAs). This catalytic function permits cleavage of transcripts complementary to piRNA guides while tolerating nucleotide mismatches—a critical adaptation for targeting rapidly evolving endogenous transposons without requiring *de novo* small RNA synthesis.^24^ The minimal sequence requirements for PIWI-mediated cleavage prevent inadvertent silencing of host RNAs, enhancing discrimination between foreign and self-RNA sequences.^24^ These proteins are indispensable for piRNA biogenesis, transposon silencing, and lineage development, with primary roles in maintaining cellular identity and supporting self-renewal processes vital for stem cell therapy and epigenetic regulation.^25^ Aberrant PIWI expression disrupts DNA methylation patterns and promotes oncogenesis through multiple mechanisms: suppression of growth inhibitors, sustained proliferation, and facilitation of cancer-driving genomic mutations.^26^ As summarized in Table 1, all four model organisms maintain germline genome stability through transposon silencing, albeit via distinct piRNA synthesis pathways and amplification mechanisms. Figure 1 provides a schematic overview to facilitate the interpretation of these comparative data. These evolutionary variations present opportunities for translational applications in human stem cell therapy. For instance, the *Drosophila* ping-pong amplification cycle could inspire self-sustaining piRNA systems to enhance silencing persistence in stem cells. Conversely, leveraging the oncogenic properties of human HIWI (PIWIL1), tissue-specific promoters could be engineered to confine PIWI expression to target tissues and prevent off-target effects.

**Table 1:** Expression of PIWI protein homologues in model organisms and related diseases.

Mouse				
PIWI congeners	Features of combined piRNA	PIWI congeners function	Related diseases	Reference(s)
PIWIL1 (MIWI)	The bound piRNAs are 30 bases long.	• Stabilizes spermatogenesis-associated mRNAs • Guides piRNA-mediated mRNA cleavage • Post-transcriptional transposon silencing regulator	Male mouse sterility	17,27
PIWIL2 (MILI)	The bound piRNAs are 26 bases in length. It can bind to two types of piRNAs generated during the pre-pachytene and pachytene stages. The MILI endonuclease domain is involved in initiating the ping-pong cycle of piRNAs.	• Transcriptional transposon silencing • Sole PIWI in female mouse reproductive cells • Essential for hippocampal neurogenesis	Sterile, senescence, anxiety	28
PIWIL4 (MIWI2)	The combined piRNAs were 28 bases long; they can bind to piRNAs generated during the pre-pachytene stages.	• Transcriptional transposon silencing	Reduced fertility	29

*Human*				

PIWI congeners	PIWI congeners function		Related diseases	Reference(s)

PIWIL1 (HIWI)	• Drives tumorigenesis and metastasis via cell migration/invasion promotion, epithelial-to-mesenchymal transition induction, and stem-like property enhancement • Apoptosis inhibition		Azoospermia, bladder cancer, breast cancer, cervical cancer, colon cancer, endometrial carcinoma, esophageal squamous cell carcinoma, glioma, hepatocellular carcinoma, ovarian cancer, renal cell carcinoma, soft tissue sarcoma, stomach cancer, *etc*.	27,30,31
PIWIL2 (HILI)	• piRNA-conjugated cancer stem cell development • Tumor-initiating cell regulation		Hepatocellular carcinoma, male infertility, oral squamous cell carcinoma, prostate cancer, *etc*.	32, 33, 34
PIWIL3 (HIWI3)	• Adult ovarian follicular expression • Pancreatic cell homeostasis maintenance • Cancer cell proliferation/migration/invasion		Breast cancer, melanoma, ovarian cancer, pancreatic cancer, lung cancer, stomach cancer, *etc*.	35
PIWIL4 (HIWI2)	• Chromatin modification • DICER-independent miRNA generation • Endogenous retrotransposon inhibition • Pancreatic cell homeostasis		AIDS, breast cancer, cervical cancer, colorectal cancer, hepatocellular carcinoma, intrahepatic bile duct carcinoma, Leydig cell tumor, soft tissue sarcoma, male azoospermia, non-small cell lung cancer, ovarian cancer, pancreatic cancer, renal cell carcinoma, testicular tumor, type 2 diabetes, *etc*.	36, 37, 38, 39

*Drosophila melanogaster*				

PIWI congeners	Features of combined piRNA	PIWI congeners function	Related diseases	Reference(s)

PIWI	The combined piRNAs are 25 bases long.	• Epigenetic modification of transposon loci and protein-coding genes	Infertility, sterility	40
Aubergine (Aub)	The combined piRNAs are 24 bases long.	• Germline stem cell regeneration/differentiation • Ping-pong cycle participation	Infertility, sterility	41
Argonaute3 (Ago3)	The combined piRNAs are 23 bases long.	• Transposon regulation • Ping-pong cycle participation	Infertility and low male reproductive capacity	42

*C. elegans*				

PIWI congeners	Features of combined piRNA	PIWI congeners function	Related diseases	Reference(s)

PRG-1	The combined piRNAs are 21 bases (21U RNA) long.	• 21U-RNA physical interaction/maturation • Transposon silencing participation	Offspring reduction, temperature-sensitive fertility defects	43

### Biogenesis and function of piRNAs

piRNAs originate predominantly from single-stranded piRNA clusters, constituting approximately 90% of all piRNAs.^44^ Designated substrates are processed into piRNA precursors that undergo nuclear export to the cytoplasm, where they experience sequential cleavage and modification events. Maturation occurs through two distinct pathways: primary and secondary processing.^45^ The primary processing pathway—operational in both somatic and germ cells—is principally facilitated by the endonuclease Zucchini (Zuc). Zuc generates a piRNA subset characterized by pronounced 5′ uridine bias.^46^ These precursor piRNAs associate with PIWI proteins through coordinated actions of heat shock protein 83 (HSP83) and Shu chaperones, forming functional PIWI/piRNA complexes. Prior to nuclear reimport, these complexes undergo 3′-terminal 2′-O-methylation by Hen1 methyltransferase, which exhibits no nucleotide specificity.^47^^,^^48^ Secondary processing, termed the ping-pong amplification cycle, requires Argonaute 3 (Ago3) and Aubergine (Aub). This mechanism features reciprocal cleavage of sense and antisense transposon transcripts: Aub cleaves sense strand precursors guided by antisense piRNAs, generating sense strand piRNAs that load onto Ago3. Conversely, Ago3-bound sense piRNAs direct cleavage of antisense transcripts, producing antisense piRNAs that subsequently associate with Aub. This amplification cycle is essential for efficient piRNA maturation and transposon silencing by PIWI-clade Argonautes.^49^^,^^50^ piRNAs serve as critical guardians of genomic stability through transposon silencing, thereby supporting stem cell functionality.^51^ Beyond gonadal tissues, piRNAs are functionally significant in non-gonadal cell types (*e.g.*, neurons), where they regulate neuronal differentiation, axonal regeneration, and specialized neural functions. Aberrant piRNA expression in these contexts is implicated in neurodegenerative pathologies, underscoring their dual importance in developmental processes and disease mechanisms.^52^

### Function of PIWI/piRNAs

PIWI/piRNA complexes form through specific association of piRNAs with PIWI proteins, executing pivotal roles in lineage specification, stem cell self-renewal, transposon silencing, and epigenetic regulation. These complexes recruit epigenetic effectors, including histone-modifying enzymes and DNA methyltransferases, to mediate transcriptional control of gene expression.^53^ At post-transcriptional levels, they silence diverse RNA targets encompassing mRNAs, pre-mRNAs, and long non-coding RNAs (lncRNAs). PIWI/piRNA pathways critically modulate key signaling cascades (PI3K/AKT, STAT, TGF-β, FAS) through dual mechanisms: transcriptional regulation of pathway components and direct targeting of signaling transcripts, thereby governing apoptosis, proliferation, and necrosis.^54^ Disruption of PIWI/piRNA signaling or DNA remethylation processes underlies severe pathologies, including male infertility, underscoring their essential role in maintaining genomic integrity in germline and stem cells. Aberrant PIWI/piRNA expression in somatic malignancies establishes their utility as diagnostic biomarkers and prognostic indicators. Their multifaceted gene regulatory functions position PIWI/piRNAs as promising therapeutic targets for innovative cancer interventions. Structural studies reveal that both the piRNA guide and PIWI’s PAZ domain mediate direct protein–protein interactions within these complexes, facilitating essential multiprotein assemblies that regulate cellular function. This scaffolding capacity significantly expands the regulatory potential of PIWI/piRNA complexes within cellular architecture.^55^^,^^56^

## Role of PIWI/piRNAs in stem cell epigenetic regulation

### Transcriptional regulation

PIWI/piRNA complexes regulate stem cell activity by targeting transcriptional regulatory factors and TEs. TEs constitute dynamic DNA sequences ubiquitous in eukaryotic genomes, classified as mobile endogenous genetic elements divided into two major categories: DNA transposons that mobilize via a “cut-and-paste” mechanism involving excision and reintegration at new genomic loci, and retrotransposons that operate through a “copy-and-paste” mechanism whereby RNA intermediates are reverse-transcribed into cDNA prior to genomic integration.^57^^,^^58^ TE insertions exhibit context-dependent consequences—potentially serving as sources of genomic innovation while posing risks through illegitimate recombination, double-stranded DNA breaks during replication, disruption of coding sequences, and aberrant expression of adjacent genes via promoter activity.^59^ Their autonomous replication enables exponential expansion, functioning as both mutational hazards and evolutionary drivers. The piRNA-mediated silencing machinery suppresses TE-induced mutagenesis while maintaining transcriptional fidelity in stem cells.^50^ A distinctive property of PIWI/piRNA complexes is their sustained molecular engagement: once bound to targets, complexes remain stably associated throughout the transcript lifecycle. This persistent silencing mechanism, coupled with PIWI’s diverse piRNA repertoire, constrains TE mobility, minimizes off-target effects, and preserves metazoan lineage integrity.^60^

**Figure 2:**
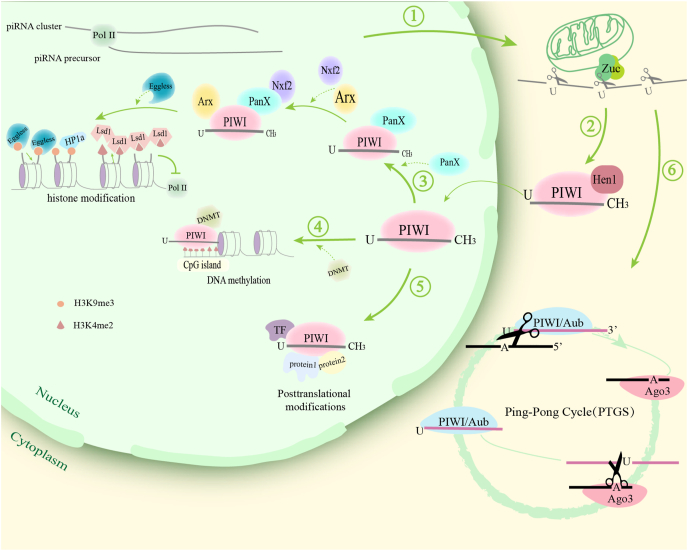
PIWI/piRNA complex: Biogenesis and epigenetic regulation mechanisms. ① piRNA clusters are transcribed into precursors, processed by Zucchini into mature 5′-U-biased piRNAs in the cytoplasm. ② Hen1 methylates piRNAs, stabilizing PIWI/piRNA complex assembly. ③ Nuclear complex recruits Panx-associated machinery: Lsd1 removes H3K4me2 to inhibit Pol II; Eggless deposits H3K9me3 to recruit HP1a for heterochromatin formation. ④ Recruitment of DNMTs for CpG island methylation. ⑤ Direct regulation of transcription factor modifications and protein localization. ⑥ Ping-Pong cycle: Piwi-Argonaute proteins cleave complementary transposon transcripts for post-transcriptional gene silencing (PTGS) degradation.

The PIWI/piRNA complex orchestrates gene regulation through three interconnected mechanisms: transcriptional gene silencing, post-transcriptional gene silencing, and post-translational modification regulation. In transcriptional gene silencing, the pathway employs DNA methylation and histone modifications. Nuclear-translocated PIWI/piRNA complexes recruit auxiliary factors including Asterix (Arx/DmGtsf1) and Panoramix (Panx), where Arx directly binds to both PIWI and retrotransposon RNAs to initiate silencing.^61^ Panx serves as the central scaffold for PIWI-directed transcriptional gene silencing, complexing with nuclear export factors dNxf2/dNxt1 to suppress transposon expression. This Panx-mediated assembly recruits silencing effectors that execute heterochromatin formation: lysine-specific demethylase 1 (LSD1) removes activating H3K4me2 marks to inhibit RNA polymerase II activity, while SET domain bifurcated histone lysine methyltransferase 1 (SETDB1) establishes repressive H3K9me3 modifications.^62^^,^^63^ Subsequent recruitment of heterochromatin protein 1α (HP1α) completes facultative heterochromatin assembly. Concurrently, PIWI/piRNA complexes direct DNA methyltransferases (DNMTs) to methylate CpG islands at promoter regions, enforcing transcriptional repression.^64^^,^^65^ At the post-transcriptional level, piRNAs execute post-transcriptional gene silencing through sequence-specific targeting of mRNAs, pre-mRNAs, and lncRNAs. The ping-pong amplification mechanism represents a specialized post-transcriptional gene silencing pathway wherein reciprocal cleavage of transposon transcripts both silences mobile elements and amplifies the piRNA pool.^66^ Beyond nucleic acid targeting, PIWI/piRNA complexes directly engage cellular proteins through dual interaction interfaces: the piRNA molecule itself and PIWI’s PAZ domain facilitate multiprotein complex assembly, modulating subcellular localization and function. This protein-interaction capacity extends to regulating transcription factors via targeted post-translational modifications,^67^ as mechanistically depicted in Figure 2.

### Chromatin modification and remodeling

piRNAs are integral to epigenetic modification in stem cells, primarily through chromatin regulation. They function to suppress retrotransposons, maintain DNA integrity, and promote repair mechanisms that enhance chromosomal resilience while reducing euchromatic gene expression induced by DNA damage.^68^ PIWI/piRNA complexes (piRISCs) play a pivotal role in silencing TE transcription by modifying chromatin states, notably through deposition of H3K9me3 marks. The PIWI/piRNA pathway is essential for TE transcriptional silencing, mediated by H3K9me3 histone modifications that recruit heterochromatin protein 1α (HP1α).^69^ Chromatin accessibility—a key consequence of PIWI/piRNA-mediated TE silencing—is regulated through two interconnected H3K9me3-dependent pathways: recruitment of histone H1 and HP1α. These pathways collectively govern chromatin compaction, enabling precise genome-wide targeting of transposons.^70^ Heterochromatin formation critical for TE silencing involves PIWI/piRNA complex deployment to complementary nascent transcripts. This coordinated action facilitates heterochromatin establishment at transposon loci genome-wide. While PIWI-mediated silencing requires heterochromatin factor recruitment, contemporary models indicate that these effectors are selectively recruited to target-bound PIWI/piRNA complexes via specialized bridging factors.^71^ In PIWI/piRNA-deficient contexts, aging somatic cells frequently exhibit heterochromatin loss essential for TE repression. This pathway protects germline and non-aging somatic cells from TE-mediated mutagenesis, suggesting potential roles in cellular immortality and identifying TEs as significant determinants of aging.^72^ PIWI/piRNA’s regulatory scope extends beyond target TEs to adjacent genomic regions, controlling expression and chromatin accessibility. System reversibility upon PIWI/piRNA loss enables dynamic heterochromatin regulation, providing flexibility during cellular adaptations. Collectively, this pathway offers profound insights into heterochromatin dynamics, TE gene control, and nuclear genome architecture, highlighting the essential complexity of piRNA-mediated chromatin regulation.^53^

### Maintaining stemness and promoting differentiation

As undifferentiated cells possessing dual capacities for differentiation and self-renewal, stem cells require stringent stability maintenance during differentiation. PIWI/piRNAs critically regulate germline stem cell (GSC) stemness and self-renewal. In *Drosophila*, GSCs residing in the ovarian anterior differentiate into gametes through maturation-triggered self-renewal.^73^ Mechanistically, PIWI may directly interact with polycomb repressive complex 2 (PRC2) components, dysregulating gene expression by limiting PRC2’s chromatin binding capacity.^74^ PRC2, an epigenetic repressor complex mediating histone methylation, is hypothesized to be sequestered in the nucleoplasm through PIWI binding, thereby attenuating PRC2-dependent transcriptional repression.^75^ Within the ovarian piRNA pathway, Aubergine (Aub) is essential for GSC self-renewal and progeny differentiation. Aub promotes self-renewal by suppressing TE-induced DNA damage and checkpoint activation, while simultaneously regulating transcription via piRNA binding in both GSCs and their differentiated descendants.^76^ PIWI further recruits the CCR4-NOT deacetylation complex to transcriptionally repress mRNAs. Aub and CCR4-NOT exhibit synergistic interactions within GSCs, collectively reinforcing self-renewal. Crucially, piRNA loading represents a fundamental requirement for Aub-mediated self-renewal; piRNA pathway inactivation disrupts meiosis and compromises subsequent germ cell development.^77^^,^^78^ The piRNA-PIWI axis similarly supports cancer stem cell maintenance, proliferation, and survival, potentially sustaining their undifferentiated state.^79^ During pluripotent stem cell differentiation, transposon silencing remains essential, with PIWI/piRNA-mediated TE suppression constituting an evolutionarily conserved mechanism linking lineage specification and pluripotent stem cell differentiation.^80^^,^^81^

## Applications and research progress of PIWI/piRNAs in stem cell therapy

### Stem cell therapy

Stem cell therapy represents an innovative and highly promising treatment modality. The application of mesenchymal stem cells, coupled with subsequent developments in embryonic stem cells and induced pluripotent stem cells, has demonstrated significant potential for developing stem cell-based therapeutic approaches for diverse diseases. Factors, including biomaterial scaffolds, growth factors, exosomes, and genes, can all influence stem cell activity and modulate therapeutic outcomes.^82^ piRNAs are highly expressed within the exosomes of bone marrow mesenchymal stem cells and are implicated in regulating bone marrow mesenchymal stem cell proliferation and osteogenic differentiation. These effects can be synergistically utilized alongside bone scaffolds for therapeutic purposes.^83^ PIWI proteins function in human somatic stem cells and are also associated with cancer development. Dysregulation of normal stem cells can lead to their transformation into cancer stem cells. Within cancer stem cells, the PIWI/piRNA pathway can enhance stemness properties. Given the inherent resistance of cancer stem cells to chemotherapy and radiotherapy, they play a crucial role in cancer metastasis and post-treatment recurrence. Consequently, targeting the piRNA-PIWI axis offers a strategy to eliminate critical subpopulations of cancer cells that are pivotal for tumor progression, treatment resistance, and relapse. Future research focusing on this axis may lead to more precise and reliable therapeutic methods.^79^^,^^84^ Specifically, PIWI proteins and certain piRNAs enhance the resistance of cancer stem cells to chemotherapeutic agents like cisplatin at the genomic level, potentially by interfering with the cell cycle. Targeting oncogenic PIWI proteins and piRNAs thus holds promise for regulating tumor growth, invasiveness, chemotherapy resistance, and anti-apoptotic mechanisms.^85^ Furthermore, transposon-based gene delivery methods have established a foundation for stem cell-related gene therapy strategies. Among these, the Sleeping Beauty (SB) and PiggyBac (PB) transposon systems are currently the most attractive platforms for achieving stable non-viral genetic modifications in primary somatic cells, as well as stem or progenitor cells. Employing transposons for the genetic modification of hematopoietic stem cells and T cells presents a promising avenue for realizing related therapeutic applications.^86^

## Disease models

### Tumors

piR-2158 acts as a transcriptional repressor of IL-11 by competing with the AP-1 transcription factor subunit FOSL1 for binding to the IL-11 promoter. This mechanism mediates cancer stemness and tumor growth via the signal transducer and activator of transcription 3 (STAT3) signaling pathway.^87^ piR-823 increases the expression of DNMTs, promoting DNA methylation at the adenomatous polyposis coli (APC) gene and activating Wnt signaling. This activation induces cancer stemness in the luminal subtype of breast cancer cells and offers a novel therapeutic strategy for suppressing tumor initiation through cancer stem cell regulation.^88^ In glioblastoma, PIWIL1 is overexpressed and is enriched in glioma stem cell-like cells (GSCs), playing a pivotal role in maintaining their self-renewal. Silencing PIWIL1 in GSCs reduces their maintenance, diminishes tumor growth, and enhances mouse survival by regulating the stem cell marker Olig2.^30^ Additionally, the overexpression of PIWIL1 in endometrial cancer cells leads to increased levels of cancer stem cell markers (*e.g.*, ALDH1, CD44, Oct4, and Nanog), indicating its active role in cancer stem cell regulation. PIWIL1 also maintains cervical cancer stem cells.^85^^,^^89^ In multiple myeloma, piR-823 expression is up-regulated. Granulocyte-myeloid-derived suppressor cells enhance the stemness of multiple myeloma stem cells through the piR-823 pathway and DNA methylation. Silencing piR-823 effectively reverses this myeloid-derived suppressor cell-induced maintenance of the stemness of multiple myeloma stem cells, thereby reducing the *in vivo* tumor burden.^90^ PIWIL1, highly expressed in myeloma cell lines and in patients with newly diagnosed multiple myeloma, shows even higher levels in refractory/relapsed multiple myeloma patients. It promotes multiple myeloma cell proliferation and imparts resistance to chemotherapeutic drugs both *in vitro* and *in vivo*. Depleting PIWIL1 significantly overcomes this resistance, offering a new therapeutic target to reverse drug resistance in multiple myeloma patients.^91^^,^^92^ The PIWI12 gene is crucial during the tumorigenesis process for the development of tumor stem cells. Homologous to the human HIWI gene, silencing the HIWI gene reduces the proliferation of lung cancer stem cells and promotes their apoptosis, thus serving as a molecular target to inhibit lung cancer stem cell growth and offering potential therapeutic value for lung cancer treatment.^93^ In acute myeloid leukemia, PIWIL4 is overexpressed and essential for the function of leukemia stem cells and acute myeloid leukemia growth, but is dispensable for healthy human hematopoietic stem cells. Depletion of PIWIL4 in acute myeloid leukemia cells down-regulates myeloid progenitor characteristics and leukemia stem cell-associated genes, up-regulates DNA damage signaling, and prevents DNA damage, replication stress, and activation of the ATR pathway in acute myeloid leukemia cells.^37^

### Bone and joint diseases

The fundamental pathogenic mechanism of osteoporosis involves an imbalance between bone formation by osteoblasts and bone resorption by osteoclasts. To address this imbalance, a key therapeutic strategy aims to induce the differentiation of bone marrow mesenchymal stem cells into bone tissue.^94^ Notably, overexpression of piR-63049 inhibits osteoblast generation from bone marrow mesenchymal stem cells, whereas knockdown of piR-63049 enhances osteoblast formation via the Wnt2b/β-catenin signaling pathway. Furthermore, *in vivo* knockdown of piR-63049 alleviates bone loss by promoting bone formation.^92^^,^^95^ The piR-36741-PIWIL4 complex positively regulates bone morphogenetic protein-2 (BMP-2) expression. This complex interacts with methyltransferase-like 3 (METTL3) and inhibits the METTL3-mediated N6-methyladenosine modification of BMP-2 mRNA transcripts. This interaction consequently reverses the inhibitory effects of piR-36741 on osteogenic differentiation and the Smad signaling pathway in bone marrow mesenchymal stem cells.^96^ During osteogenic differentiation, piR-36741 expression is up-regulated. Silencing piR-36741 significantly impairs osteogenic differentiation, leading to reduced expression of osteogenic markers, diminished osteoblast-specific phenotypes, and decreased matrix mineralization. Conversely, administration of piR-36741 alleviates osteoporosis induced by ovariectomy in mice.^97^

The significant variations in piRNA expression observed during bone marrow mesenchymal stem cell differentiation highlight their potential as therapeutic targets for bone-related diseases, opening new avenues for therapeutic intervention.

### Other related diseases

Research demonstrates that neural stem cells in mice release exosomes/microvesicles containing piRNAs that contribute to antiviral immunity. Specifically, knockout of the piRNA-binding protein PIWIL2 in hypothalamic neural stem cells diminishes both innate and induced antiviral responses against SARS-CoV-2.^98^ The piRNA pathway is essential for maintaining homeostasis and determining the fate of hippocampal neural stem cells. In the postnatal mouse hippocampus, both MILI protein and piRNA expression are notably enriched in neural progenitor cells, with piRNA functionality being crucial for neurogenesis. Furthermore, inhibition of the piRNA pathway in adult neural progenitor cells leads to increased age-related inflammation and the generation of reactive astrocytes—conditions associated with age-related neurogenic decline and neurodegeneration.^99^ Within the ovarian GSC niche of *Drosophila*, an age-related decline in PIWI expression not only suppresses retrotransposons but also results in GSC loss, suggesting its potential as a therapeutic target for age-related tissue degeneration.^100^ The role of PIWI proteins presents greater complexity, as mutations disrupting PIWI function impair GSC self-renewal. Consequently, females harboring PIWI mutations fail to complete oogenesis, adversely affecting fertility.^40^ These insights into piRNA and PIWI proteins highlight their potential for developing interventions targeting neurodegenerative diseases and reproductive health issues related to aging and viral infections.

## Discussion

piRNAs play critical roles in the pathogenesis of various diseases; however, their functions in stem cells and somatic cells remain controversial and contradictory. First, PIWI/piRNA complexes were initially thought to be exclusively present in germ cells,^101^ but subsequent studies have revealed their regulatory functions in somatic cells, including cancer cells.^79^ Second, the essentiality of PIWI/piRNA for female fertility is debated. Some studies indicate that silencing PIWI proteins primarily affects male reproductive function, while others demonstrate that silencing PIWI/piRNA leads to oocyte developmental arrest, embryonic developmental arrest, and even death.^102^ Third, the epigenetic mechanisms of PIWI/piRNA complexes differ between GSCs and somatic cells. In GSCs, PIWI/piRNA directly recruits HP1a to mediate heterochromatin formation.^103^ In contrast, maternal PIWI deposits transient H3K9me3 marks in somatic nuclei to silence the roo transposon.^104^ In mammalian somatic cells, PIWI overexpression primarily regulates genes through post-transcriptional cleavage rather than chromatin remodeling.^105^ Lastly, PIWI/piRNA may exhibit dual roles in diseases across germline and somatic contexts. Generally, PIWI/piRNA silences transposons to prevent mutation accumulation in germ cells, maintains genomic stability, and primarily acts as a tumor suppressor. In somatic cells, however, their functions may be conflicting.^79^

## Ethical considerations and future directions

Although the PIWI/piRNA complex presents promising novel approaches for stem cell therapy across diverse diseases, significant uncertainties and technical hurdles persist in its practical application. The clinical translation of PIWI/piRNA-based therapies faces significant challenges, including inadequate targeting precision, low delivery efficiency, and complex mechanisms of action. A critical concern arises when PIWI/piRNA complexes are delivered *in vivo* (*e.g.*, via nanozyme carriers), as they may preferentially target germ cells, posing risks of unintentional germline genome editing.^21^^,^^106^ Furthermore, current ethical frameworks lack specific guidelines addressing piRNAs and their associated epigenetic modifications. Mechanistically, the inherent specificity limitations of piRNAs present substantial hurdles: unlike siRNAs, piRNAs do not require perfect sequence complementarity for target binding, compromising their precision. Additionally, as single-stranded RNAs carrying a negative charge, piRNAs exhibit significantly lower stability compared with double-stranded RNAs such as siRNAs, further complicating therapeutic development. Compounding these challenges, piRNAs engage in complex multi-layered regulatory mechanisms: they not only degrade target mRNAs but also induce histone modifications and DNA methylation. This broad epigenetic influence risks provoking extensive and potentially undesirable gene silencing effects, consequently increasing the risk of off-target side effects relative to siRNA-based approaches.^107^

Moreover, although PIWI/piRNA complexes regulate DNA methylation with context-dependent pro-apoptotic or anti-apoptotic outcomes, the precise mechanistic underpinnings of these dichotomous effects remain poorly characterized. To advance clinical translation, future research must prioritize enhancing piRNA therapeutic specificity and stability while mitigating associated side effects and biotoxicity risks. Progress in regenerative medicine and tissue engineering offers promising avenues to address delivery challenges through the integration of functionalized biomaterials. Nevertheless, clinical applications of PIWI/piRNA therapies remain scarce, underscoring substantial translational hurdles that must be overcome before routine implementation becomes feasible. Furthermore, nanozymes utilized as delivery vehicles for piRNAs present significant inherent challenges, including suboptimal solubility, unpredictable metabolic profiles, and intrinsic toxicity, all of which may adversely impact therapeutic efficacy.^106^

To overcome these limitations, future research should prioritize comprehensive solutions. Firstly, fundamental investigations into piRNA regulatory mechanisms require deepening. Simultaneously, nanocarriers co-administered with piRNA-modulating stem cells must incorporate stringent germline exclusion mechanisms while enhancing delivery precision and cell-type specificity. Secondly, leveraging AI platforms to identify disease-critical piRNAs and predict high-affinity sequences will improve targeting accuracy and therapeutic efficiency. Furthermore, developing CRISPR-PIWI fusion editors to silence transposons through epigenetic reprogramming could extend functional PIWI/piRNA activity. Additionally, establishing clear germline editing risk frameworks, strengthening regulatory policies, and implementing comprehensive operational guidelines remain essential.

## Conclusion

Recent advancements in piRNA research have significantly advanced our understanding of their biogenesis, fundamental characteristics, and roles in epigenetic regulation within stem cells. The PIWI/piRNA pathway has emerged as a major research focus due to its involvement in disease pathogenesis and therapeutic potential for stem cell-based therapies, particularly through DNA methylation and other epigenetic mechanisms. Consequently, PIWI/piRNAs are increasingly recognized as promising candidates for developing novel treatment strategies. Although numerous preclinical and early translational studies have demonstrated clear therapeutic potential, no established clinical applications targeting PIWI currently exist. This gap may stem from limitations in delivery systems, functional and mechanistic complexities, and the absence of defined regulatory standards. Future research should prioritize overcoming current limitations in PIWI/piRNA therapies by improving clinical efficacy through deeper mechanistic insights and advancing clinical trials. These efforts will ensure safe, precise therapeutic applications. The translation from theoretical frameworks to tangible clinical utility will require continued elucidation of PIWI/piRNAs' complex biological mechanisms. Fully realizing their therapeutic potential necessitates rigorous testing, validation, and development of targeted therapies.

## CRediT authorship contribution statement

**Ying Yang:** Writing – original draft. **Xiangping Luo:** Writing – review & editing. **Ermao Li:** Writing – review & editing, Conceptualization. **Zhengmao Li:** Writing – review & editing, Conceptualization. **Feiyan Zou:** Writing – review & editing. **Jiayang Liao:** Writing – review & editing. **Yizhi Wu:** Writing – review & editing. **Bo Wei:** Writing – review & editing.

## Funding

This work was supported by the Key Fund Project of Hunan Provincial Department of Education (China) (No. 23A0340), the General Guidance Project of Hunan Provincial Health Commission of China (No. 202104072014), the Hunan University Students Innovation and Entrepreneurship Training Program Project (China) (No. D202405231300501291, D202405232303034450), the Hunan Provincial Natural Science Foundation (China) (No. 2023JJ50098), and the 10.13039/501100001809National Natural Science Foundation of China (No. 82303122).

## Conflict of interests

The authors declared no competing interests.
